# Supplementary material for: Crawling and Gliding: A Computational Model for Shape-Driven Cell Migration
Source: PLoS Comput Biol. 2015 Oct 21;11(10):e1004280. doi: 10.1371/journal.pcbi.1004280 (PMC4619082; doi:10.1371/journal.pcbi.1004280)
Supplement: S1 Code — (ZIP) [file pcbi.1004280.s012.zip › release/tst/doc/html/cell_8h_source.html]

Tissue Simulation Toolkit: cell.h Source File


Tissue Simulation Toolkit
 0.1.4.1


- Main Page
- Namespaces
- Classes
- Files

- File List
- File Members

cell.h

Go to the documentation of this file.

1 /\*

2

3 Copyright 1996-2006 Roeland Merks

4

5 This file is part of Tissue Simulation Toolkit.

6

7 Tissue Simulation Toolkit is free software; you can redistribute

8 it and/or modify it under the terms of the GNU General Public

9 License as published by the Free Software Foundation; either

10 version 2 of the License, or (at your option) any later version.

11

12 Tissue Simulation Toolkit is distributed in the hope that it will

13 be useful, but WITHOUT ANY WARRANTY; without even the implied

14 warranty of MERCHANTABILITY or FITNESS FOR A PARTICULAR PURPOSE.

15 See the GNU General Public License for more details.

16

17 You should have received a copy of the GNU General Public License

18 along with Tissue Simulation Toolkit; if not, write to the Free

19 Software Foundation, Inc., 51 Franklin St, Fifth Floor, Boston, MA

20 02110-1301 USA

21

22 \*/

23 #ifndef \_CELL\_HH\_

24 #define \_CELL\_HH\_

25

26 #include "parameter.h"

27 //#define EMPTY -1

28 #include <math.h>

29

30 extern Parameter par;

31 class Dish;

32

33 class Cell

34 {

35

36  friend class Dish;

37  friend class CellularPotts;

38  friend class Info;

39

40 public:

46  Cell(const Dish &who, int

47  settau=1) {

48

49  owner=&who;

50  ConstructorBody(settau);

51  }

52

53  Cell(void) {

54  chem = new double[par.n\_chem];

55  };

56

57  ~Cell(void);

58

59

61  Cell(const Cell &src) {

62

63  // make an exact copy (for internal use)

64  sigma=src.sigma;

65  amount++;

66  area=src.area;

67  target\_area=src.target\_area;

68  length=src.length;

69  target\_length=src.target\_length;

70  growth\_threshold=src.growth\_threshold;

71  mother=src.mother;

72  daughter=src.daughter;

73  times\_divided=src.times\_divided;

74  date\_of\_birth=src.date\_of\_birth;

75  colour\_of\_birth=src.colour\_of\_birth;

76  tau=src.tau;

77  alive=src.alive;

78  v[0]=src.v[0];

79  v[1]=src.v[1];

80  n\_copies=src.n\_copies;

81  sum\_x=src.sum\_x;

82  sum\_y=src.sum\_y;

83  sum\_xx=src.sum\_xx;

84  sum\_yy=src.sum\_yy;

85  sum\_xy=src.sum\_xy;

86  owner=src.owner;

87

88  chem = new double[par.n\_chem];

89  for (int ch=0;ch<par.n\_chem;ch++)

90  chem[ch]=src.chem[ch];

91

92

93  }

94

103  void CellBirth(Cell &mother);

104

111  inline Cell &operator=(const Cell &src) {

112  colour=src.colour;

113  alive=src.alive;

114  sigma=src.sigma;

115  area=src.area;

116  tau=src.tau;

117  target\_area=src.target\_area;

118  v[0]=src.v[0];

119  v[1]=src.v[1];

120  n\_copies=src.n\_copies;

121

122  sum\_x=src.sum\_x;

123  sum\_y=src.sum\_y;

124  sum\_xx=src.sum\_xx;

125  sum\_yy=src.sum\_yy;

126  sum\_xy=src.sum\_xy;

127

128  length=src.length;

129  target\_length=src.target\_length;

130  amount++;

131  owner=src.owner;

132

133  chem = new double[par.n\_chem];

134  for (int ch=0;ch<par.n\_chem;ch++)

135  chem[ch]=src.chem[ch];

136

137  return \*this;

138

139  }

140

142  inline bool AliveP(void) const {

143  return alive;

144  }

145

147  inline int Colour(void) const {

148

149  /\* if (par.dynamicJ)

150  return colour;

151  else \*/

152  return tau+1;

153

154  };

155

157  inline void setTau(int settau) {

158  tau=settau;

159  }

160

162  inline int getTau(void) {

163  return tau;

164  }

166  inline int SetColour(const int new\_colour) {

167  return colour=new\_colour;

168  }

169

170  /\* \brief Returns the energy between this cell and cell2.

171

172  Called from CellularPotts::DeltaH.

173  \*\*/

174  int EnergyDifference(const Cell &cell2) const;

175

177  inline int Area() const {

178  return area;

179  }

180

182  inline int TargetArea() const {

183  return target\_area;

184  }

185

190  inline double TargetLength() const {

191  return target\_length;

192  }

193

195  inline double SetTargetLength(double l) {

196  return target\_length=l;

197  }

198

199

201  inline void PrintInertia(void) {

202

203  double ixx=(double)sum\_xx-(double)sum\_x\*sum\_x/(double)area;

204  double iyy=(double)sum\_yy-(double)sum\_y\*sum\_y/(double)area;

205  double ixy=(double)sum\_xy-(double)sum\_x\*sum\_y/(double)area;

206

207  cerr << "ixx = " << ixx << "\n";

208  cerr << "iyy = " << iyy << "\n";

209  cerr << "ixy = " << ixy << "\n";

210

211  }

212

213  // return the current length

214  inline double Length(void) {

215  return length;

216  }

217

225  static void ClearJ(void);

226  double polarvec[9];

227  void RenormPolarVec(void);

228

233  static inline int MaxSigma() {

234  return maxsigma;

235  }

236

238  inline int Sigma() const {

239  return sigma;

240  }

241

243  inline int SetTargetArea(const int new\_area) {

244  return target\_area=new\_area;

245  }

246

248  inline void Apoptose() {

249  alive=false;

250  }

251

253  inline int IncrementTargetArea() {

254  return ++target\_area;

255  }

256

258  inline int DecrementTargetArea() {

259  return --target\_area;

260  }

261

263  inline int Mother(void) const { return mother; }

264

266  inline int Daughter(void) const { return daughter; }

267

269  inline int TimesDivided(void) const { return times\_divided; }

270

272  inline int DateOfBirth(void) const { return date\_of\_birth; }

273

275  inline int ColourOfBirth(void) const { return colour\_of\_birth; }

276

278  inline int GetJ(const Cell &c2) const {

279  return J[sigma][c2.sigma];

280  }

281

282

284  inline static int SetJ(int t1,int t2, int val) {

285  return J[t2][t1]=J[t1][t2]=val;

286  }

287

288

289  // Deal with gradient measurements:

290

292  inline double\* SetGrad(double \*g) {

293  grad[0]=g[0];

294  grad[1]=g[1];

295  return grad;

296  }

297

299  inline const double\* GetGrad(void) const {

300  return grad;

301  }

302

304  inline const double GradX() const {

305  return grad[0];

306  }

307

309  inline const double GradY() const {

310  return grad[1];

311  }

312

314  inline double\* AddToGrad(double \*g) {

315  grad[0]+=g[0];

316  grad[1]+=g[1];

317  return grad;

318  }

319

321  inline void ClearGrad(void) {

322  grad[0]=0.;

323  grad[1]=0.;

324  }

325

329  void MeasureCellSize(Cell &c);

330

331 private:

336  static void ReadStaticJTable(const char \*fname);

337

338  // used internally by dish in "CellGrowthAndDivision"

339  inline int GrowthThreshold(void) const { return growth\_threshold; }

340

341  // used internally by class CellularPotts

342  inline void CleanMoments(void) {

343  sum\_x = sum\_y = sum\_xx = sum\_xy = sum\_yy = area = target\_area = 0;

344  }

345  // used internally by class CellularPotts

346  inline double AddSiteToMoments(int x,int y, double new\_l=-1. ) {

347

348  // Add a site to the raw moments, then update and return the

349  // length of the cell

350

351  // sum\_x, sum\_y, sum\_xx, sum\_xy and sum\_yy are adjusted

352  // Eventually this function may be used to carry

353  // out all necessary adminstration at once

354  sum\_x+=x;

355  sum\_y+=y;

356  sum\_xx+=x\*x;

357  sum\_yy+=y\*y;

358  sum\_xy+=x\*y;

359

360  // update length (see appendix. A, Zajac.jtb03), if length is not given

361  // NB. 24 NOV 2004. Found mistake in Zajac's paper. See remarks in

362  // method "Length(..)".

363  if (new\_l<0.) {

364  length=Length(sum\_x,sum\_y,sum\_xx,sum\_yy,sum\_xy,area);

365  } else {

366  length=new\_l;

367  }

368  return length;

369  }

370

371  // used internally by class CellularPotts

372  inline double RemoveSiteFromMoments(int x,int y, double new\_l=-1.) {

373

374  // Remove a site from the raw moments, then update and return the

375  // length of the cell

376

377  // sum\_x, sum\_y, sum\_xx, sum\_xy and sum\_yy are adjusted

378  // Eventually this function may be used to carry

379  // out all necessary adminstration at once

380  sum\_x-=x;

381  sum\_y-=y;

382  sum\_xx-=x\*x;

383  sum\_yy-=y\*y;

384  sum\_xy-=x\*y;

385

386  // update length (see app. A, Zajac.jtb03), if length is not given

387  if (new\_l<0.) {

388  length=Length(sum\_x,sum\_y,sum\_xx,sum\_yy,sum\_xy,area);

389  } else {

390  length=new\_l;

391  }

392  return length;

393  }

394

396  //components (used internally)

397  inline double Length(long int s\_x,long int s\_y,long int s\_xx,

398  long int s\_yy,long int s\_xy,long int n) {

399

400  // inertia tensor (constructed from the raw momenta, see notebook)

401  double iyy=(double)s\_xx-(double)s\_x\*s\_x/(double)n;

402  double ixx=(double)s\_yy-(double)s\_y\*s\_y/(double)n;

403  double ixy=-(double)s\_xy+(double)s\_x\*s\_y/(double)n;

404

405  double rhs1=(ixx+iyy)/2., rhs2=sqrt( (ixx-iyy)\*(ixx-iyy)+4\*ixy\*ixy )/2.;

406

407  double lambda\_b=rhs1+rhs2;

408  //double lambda\_a=rhs1-rhs2;

409

410  // according to Zajac et al. 2003:

411  //return 2\*sqrt(lambda\_b);

412  // Grumble, this is not right!!!

413  // Must divide by mass!!!!!!

414

415  // see: http://scienceworld.wolfram.com/physics/MomentofInertiaEllipse.html

416  // cerr << "n = " << n << "\n";

417  return 4\*sqrt(lambda\_b/n);

418

419  // 2\*sqrt(lambda\_b/n) give semimajor axis. We want the length.

420

421  }

422

423  // return the new length that the cell would have

424  // if site (x,y) were added.

425  // used internally by CellularPotts

426  inline double GetNewLengthIfXYWereAdded(int x, int y) {

427

428  return Length(sum\_x+x,sum\_y+y,

429  sum\_xx+x\*x,sum\_yy+y\*y,sum\_xy+x\*y,area+1);

430

431  }

432

433  // return the new length that the cell would have

434  // if site (x,y) were removed

435  // used internally by CellularPotts

436  inline double GetNewLengthIfXYWereRemoved(int x, int y) {

437

438  return Length(sum\_x-x,sum\_y-y,

439  sum\_xx-x\*x,sum\_yy-y\*y,sum\_xy-x\*y,area-1);

440

441  }

442

443

444 private:

446  inline int IncrementArea() {

447  return ++area;

448  }

449

451  inline int DecrementArea() {

452  return --area;

453  }

454

455

460  inline int SetAreaToTarget(void) {

461  return area=target\_area;

462  }

463

465  void ConstructorBody(int settau=1);

466

467  // returns the maximum cell type index

468  // (depends on Jtable)

469  static int MaxTau(void) {

470  return maxtau;

471  }

472

473 protected:

474  int colour;

475  bool alive;

476  int sigma; // cell identity, 0 if medium

477  int tau; // Cell type, when dynamicJ's are not used

478

479  // Two dimensional (square) array of ints, containing the J's.

480  double length; // length of the cell;

481  double target\_length;

482

483  // Dynamically increased when cells are added to the system

484  // unless a static Jtable is used (currently this is the default situation)

485  static int \*\*J;

486  static int maxtau;

487

488  // Amount: the number of Cell instantations, INCLUDING copies

489  // For internal use only.

490  // Reading amount is NOT the way to get the number of cells!!

491  static int amount;

492  static int capacity;

493  static int maxsigma; // the last cell identity number given out

494

495

496  // indices of mother and daughter

497  // (Note: no pointers, cells may be relocated)

498  int mother;

499  int daughter;

500  int times\_divided;

501  int date\_of\_birth;

502  int colour\_of\_birth;

503

504  int area;

505  int target\_area;

506  int growth\_threshold;

507

508  double v[2];

509  int n\_copies; // number of expansions of this cell

510  // gradient of a chemical (to be extended to the total number chemicals)

511  double grad[2];

512

513  double \*chem;

514  // Raw moments of the cells

515  // Are used to calculate minor and major axes

516  // and center of mass

517  // are locally adjusted, so axes are easily

518  // and quickly calculated!

519

520  // N.B: N is area!

521

522  long int sum\_x;

523  long int sum\_y;

524  long int sum\_xx;

525  long int sum\_yy;

526  long int sum\_xy;

527

528  const Dish \*owner; // pointer to owner of cell

529

530 };

531

532 #endif

Cell::amount

static int amount

**Definition:** cell.h:491

Cell::MeasureCellSize

void MeasureCellSize(Cell &c)

Cell::ColourOfBirth

int ColourOfBirth(void) const

Returns the cell type at the time of birth.

**Definition:** cell.h:275

Cell

**Definition:** cell.h:33

Cell::times\_divided

int times\_divided

**Definition:** cell.h:500

Cell::growth\_threshold

int growth\_threshold

**Definition:** cell.h:506

Cell::setTau

void setTau(int settau)

Set cell type of this Cell.

**Definition:** cell.h:157

Cell::operator=

Cell & operator=(const Cell &src)

Assignment operator.

**Definition:** cell.h:111

Cell::EnergyDifference

int EnergyDifference(const Cell &cell2) const

**Definition:** cell.cpp:180

Cell::Cell

Cell(const Cell &src)

Default copy constructor.

**Definition:** cell.h:61

Cell::Daughter

int Daughter(void) const

Cell lineage tracking: get the cell's daughter.

**Definition:** cell.h:266

Cell::sum\_xx

long int sum\_xx

**Definition:** cell.h:524

Cell::owner

const Dish \* owner

**Definition:** cell.h:528

Cell::grad

double grad[2]

**Definition:** cell.h:511

Cell::Sigma

int Sigma() const

Returns the cell's cell identity number.

**Definition:** cell.h:238

Cell::GetJ

int GetJ(const Cell &c2) const

Returns the bond energy J between this cell and cell c2.

**Definition:** cell.h:278

Cell::n\_copies

int n\_copies

**Definition:** cell.h:509

Cell::TimesDivided

int TimesDivided(void) const

Returns a counter keeping track of the number of divisions.

**Definition:** cell.h:269

CellularPotts

**Definition:** ca.h:57

Cell::GradX

const double GradX() const

Returns the cell's measured gradient. Currently not in use.

**Definition:** cell.h:304

Parameter::n\_chem

int n\_chem

**Definition:** parameter.h:49

Cell::SetJ

static int SetJ(int t1, int t2, int val)

Sets bond energy J between cell type t1 and t2 to val.

**Definition:** cell.h:284

Cell::TargetArea

int TargetArea() const

Return Cell's target area.

**Definition:** cell.h:182

Cell::J

static int \*\* J

**Definition:** cell.h:485

Cell::CellBirth

void CellBirth(Cell &mother)

Add a new cell to the dish.

**Definition:** cell.cpp:66

Cell::maxtau

static int maxtau

**Definition:** cell.h:486

Cell::area

int area

**Definition:** cell.h:504

Cell::Area

int Area() const

Return Cell's actual area.

**Definition:** cell.h:177

Cell::getTau

int getTau(void)

Get cell type of this Cell.

**Definition:** cell.h:162

Cell::ClearJ

static void ClearJ(void)

Clears the table of J's.

**Definition:** cell.cpp:192

Cell::Cell

Cell(const Dish &who, int settau=1)

Constructor to insert a cell into Dish "who".

**Definition:** cell.h:46

Cell::target\_area

int target\_area

**Definition:** cell.h:505

Cell::MaxSigma

static int MaxSigma()

Returns the maximum cell identity number in the Dish. This would normally be the number of cells in t...

**Definition:** cell.h:233

Cell::SetGrad

double \* SetGrad(double \*g)

Set the current gradient of the cell to g. Currently not in use.

**Definition:** cell.h:292

Cell::Apoptose

void Apoptose()

Sends the current cell into apoptosis.

**Definition:** cell.h:248

Dish

The virtual Petri dish.

**Definition:** dish.h:43

Parameter

**Definition:** parameter.h:28

Cell::sum\_yy

long int sum\_yy

**Definition:** cell.h:525

Cell::daughter

int daughter

**Definition:** cell.h:499

Cell::GradY

const double GradY() const

Returns the cell's measured gradient. Currently not in use.

**Definition:** cell.h:309

Cell::IncrementTargetArea

int IncrementTargetArea()

Decrement the cell's target area by one unit.

**Definition:** cell.h:253

Cell::chem

double \* chem

**Definition:** cell.h:513

Cell::sigma

int sigma

**Definition:** cell.h:476

Cell::RenormPolarVec

void RenormPolarVec(void)

Cell::colour\_of\_birth

int colour\_of\_birth

**Definition:** cell.h:502

Cell::Length

double Length(void)

**Definition:** cell.h:214

Cell::sum\_xy

long int sum\_xy

**Definition:** cell.h:526

Cell::DecrementTargetArea

int DecrementTargetArea()

Increment the cell's target area by one unit.

**Definition:** cell.h:258

Cell::colour

int colour

**Definition:** cell.h:474

Cell::SetTargetArea

int SetTargetArea(const int new\_area)

Sets the target area of the cell.

**Definition:** cell.h:243

Cell::Colour

int Colour(void) const

Returns the cell colour.

**Definition:** cell.h:147

Cell::capacity

static int capacity

**Definition:** cell.h:492

Cell::sum\_y

long int sum\_y

**Definition:** cell.h:523

Cell::polarvec

double polarvec[9]

**Definition:** cell.h:226

Cell::alive

bool alive

**Definition:** cell.h:475

Cell::v

double v[2]

**Definition:** cell.h:508

Cell::date\_of\_birth

int date\_of\_birth

**Definition:** cell.h:501

Cell::TargetLength

double TargetLength() const

Return Cell's target length.

**Definition:** cell.h:190

Cell::sum\_x

long int sum\_x

**Definition:** cell.h:522

Cell::length

double length

**Definition:** cell.h:480

par

Parameter par

**Definition:** parameter.cpp:210

Cell::target\_length

double target\_length

**Definition:** cell.h:481

Cell::ClearGrad

void ClearGrad(void)

Currently not in use (remove?)

**Definition:** cell.h:321

Cell::Cell

Cell(void)

**Definition:** cell.h:53

Cell::mother

int mother

**Definition:** cell.h:498

Cell::DateOfBirth

int DateOfBirth(void) const

Returns Monte Carlo Step (MCS) when this cell originated.

**Definition:** cell.h:272

Cell::GetGrad

const double \* GetGrad(void) const

Returns the cell's measured gradient. Currently not in use.

**Definition:** cell.h:299

Cell::AliveP

bool AliveP(void) const

Returns false if Cell has apoptosed (vanished).

**Definition:** cell.h:142

Cell::maxsigma

static int maxsigma

**Definition:** cell.h:493

Cell::SetTargetLength

double SetTargetLength(double l)

Set the Cell's target length.

**Definition:** cell.h:195

Cell::tau

int tau

**Definition:** cell.h:477

Cell::SetColour

int SetColour(const int new\_colour)

Set color of this cell to new\_colour, irrespective of type.

**Definition:** cell.h:166

Cell::PrintInertia

void PrintInertia(void)

Debugging function used to print the cell's current inertia tensor (as used for calculations of the l...

**Definition:** cell.h:201

Cell::Mother

int Mother(void) const

Cell lineage tracking: get the cell's parent.

**Definition:** cell.h:263

parameter.h

Cell::AddToGrad

double \* AddToGrad(double \*g)

Currently not in use (remove?)

**Definition:** cell.h:314

Info

Enables interactive querying of the simulation.

**Definition:** info.h:36

Cell::~Cell

~Cell(void)

**Definition:** cell.cpp:52


---

Generated on Thu Aug 14 2014 22:04:01 for Tissue Simulation Toolkit by  

 1.8.6
